# Leveraging explainable machine learning to identify gait biomechanical parameters associated with anterior cruciate ligament injury

**DOI:** 10.1038/s41598-022-10666-2

**Published:** 2022-04-22

**Authors:** Christos Kokkotis, Serafeim Moustakidis, Themistoklis Tsatalas, Charis Ntakolia, Georgios Chalatsis, Stylianos Konstadakos, Michael E. Hantes, Giannis Giakas, Dimitrios Tsaopoulos

**Affiliations:** 1 Institute for Bio-Economy & Agri-Technology, Center for Research and Technology Hellas, 38333 Vólos, Greece; 2 TEFAA, Department of Physical Education & Sport Science, University of Thessaly, 42100 Trikala, Greece; 3 AIDEAS OÜ, 10117 Tallinn, Estonia; 4 Hellenic National Center of COVID-19 Impact on Youth, University Mental Health Research Institute, 11527 Athens, Greece; 5 School of Naval Architecture and Marine Engineering, National Technical University of Athens, 15772 Athens, Greece; 6 Department of Orthopaedic Surgery and Musculoskeletal Trauma, Faculty of Medicine, School of Health Sciences, University General Hospital of Larissa, 41110 Larissa, Greece; 7 Animus, 41500 Larissa, Greece

**Keywords:** Computational biology and bioinformatics, Health care

## Abstract

Anterior cruciate ligament (ACL) deficient and reconstructed knees display altered biomechanics during gait. Identifying significant gait changes is important for understanding normal and ACL function and is typically performed by statistical approaches. This paper focuses on the development of an explainable machine learning (ML) empowered methodology to: (i) identify important gait kinematic, kinetic parameters and quantify their contribution in the diagnosis of ACL injury and (ii) investigate the differences in sagittal plane kinematics and kinetics of the gait cycle between ACL deficient, ACL reconstructed and healthy individuals. For this aim, an extensive experimental setup was designed in which three-dimensional ground reaction forces and sagittal plane kinematic as well as kinetic parameters were collected from 151 subjects. The effectiveness of the proposed methodology was evaluated using a comparative analysis with eight well-known classifiers. Support Vector Machines were proved to be the best performing model (accuracy of 94.95%) on a group of 21 selected biomechanical parameters. Neural Networks accomplished the second best performance (92.89%). A state-of-the-art explainability analysis based on SHapley Additive exPlanations (SHAP) and conventional statistical analysis were then employed to quantify the contribution of the input biomechanical parameters in the diagnosis of ACL injury. Features, that would have been neglected by the traditional statistical analysis, were identified as contributing parameters having significant impact on the ML model’s output for ACL injury during gait.

## Introduction

Anterior cruciate ligament (ACL) tear is a frequent knee injury occurring in young active individuals during sport activities like basketball, football, ski and volleyball^1,2^. The primary function of the ACL is to confine excessive posterior translation and external rotation of the femur relatively to the tibia against forces that act on the joint during gait and other activities^3–6^. As a result, an ACL deficient knee presents significant reflect on joint stability and biomechanics^7–9^. Studies utilising three-dimensional (3D) motion analysis have shown altered joint motion in ACL deficient knees during daily activities, such as walking, ascending and descending stairs or jumping^10–12^. This deviation causes a shift on the contact area and magnitude of shear forces at the knee joint which can lead to the initiation of osteoarthritis^13–16^.

ACL reconstruction (ACLR) aims to lessen these changes in knee biomechanics. Annually 130.000 ACL reconstruction surgeries are performed in United States^17^. Although ACLR provides an improvement in knee stability and kinematics it is still questionable if the results are equal to pre-injury standards^18,19^. As it was observed in several studies, increase or decrease in peak external knee-adduction moment, peak internal-rotation angle, increased medial contact force and decreased knee flexion angles were related to knee-joint cartilage loading and degeneration^13,20–22^. Reductions in peak knee-flexion angle and external knee-flexion moment during the loading phase of gait have been reported at 6 to 60 months after ACLR^23–25^.

Machine learning (ML) is an artificial intelligence (AI) analytic tool that constructs algorithms to identify patterns and characteristics contained within datasets. The goal is to train and validate prediction algorithms to achieve a desired result^26^. Musculoskeletal-specific models have already been developed to identify and classify fractures and predict functional outcomes after primary total knee arthroplasty (TKA)^27^. In 2017, Olczak et al. used deep learning techniques based on medical imaging to examine the feasibility of using AI to identify fractures in skeletal radiographs^28^. In another study, Kunze et al. based on partially modifiable risk factors developed ML algorithms to predict dissatisfaction after TKA^29^. Recent studies with individual-level datasets of gait analyses from kinetic skeletal tracking and advanced MR imaging (MRI) techniques focused on the determination of early progression of knee osteoarthritis (KOA)^30^. Moustakidis et al. proposed a novel fuzzy decision tree-based support vector machine (SVM) classifier by using 3-D ground reaction force (GRF) measurements to investigate KOA severity and to distinguish between asymptotic and osteoarthritis knee gait patterns^31^. Furthermore, Pedoia et al. performed ML multidimensional data analysis by using MR imaging and biomechanical data^32^. They demonstrated that the analysis potentially indicates that cartilage composition may be an imaging biomarker for early KOA.

Machine learning approaches have been also used in studies to identify ACL injury based on MRI and biomechanical data or ACLR gait patterns with the aid of motion sensors. In 2017, Mazlan et al. proposed an ACL injury diagnosis system by using ACL injury MRI (normal, partial and crucial ACL) and SVM algorithm^33^. In another study, Chang et al. used MRI and deep learning techniques for the detection of complete ACL tear and achieved 96% test set accuracy^34^. Furthermore, Christian et al. used gait kinematics and ML techniques (SVM) to develop a pattern recognition system for diagnosis and evaluation of therapeutic treatment effect^35^. In another study, Zeng et al. proposed an approach for detection of the presence of ACL injury using kinematic features and neural networks^36^. Moreover, Todesco et al. proposed an ML approach for the identification of ACL gait patterns based on motion sensors data for on the field activities in rugby players^37^.

Despite the relatively large number of ML studies on the field of ACL, the reported trained ML models are treated as black boxes. The lack of transparency and explainability of the models result to poor understanding of their inner workings and the rationale behind their decision-making mechanism. This paper focuses on the development of an explainable ML-empowered methodology to identify important biomechanical parameters associated with ACL injury. The aims of this study are: (i) to estimate the feature importance in the classification process and examine how much each of the features contributed to the final ML decisions and (ii) to investigate differences in sagittal plane kinematics and kinetics of the gait cycle between different patient groups based on a novel approach that combines explainable ML and statistical analytics. To achieve these goals, an extensive experimental setup was designed including biomechanical data collection, a thorough comparative analysis with seven well-known classifiers and a state-of-the-art explainability analysis.

## Results

### Comparative analysis

The proposed ML pipeline was initially applied on the three-class problem in which the patient groups CON, ACLD and ACLR are considered as separate classes. The proposed FS technique was executed on the pre-processed version of the 3-class dataset ranking the available features with respect to their relevance. The ML models were trained on feature subsets of increasing dimensionality (with a step of 1) and the testing classification accuracies were finally calculated until the full feature set has been tested. The classification results are given below.

**Figure 1:**
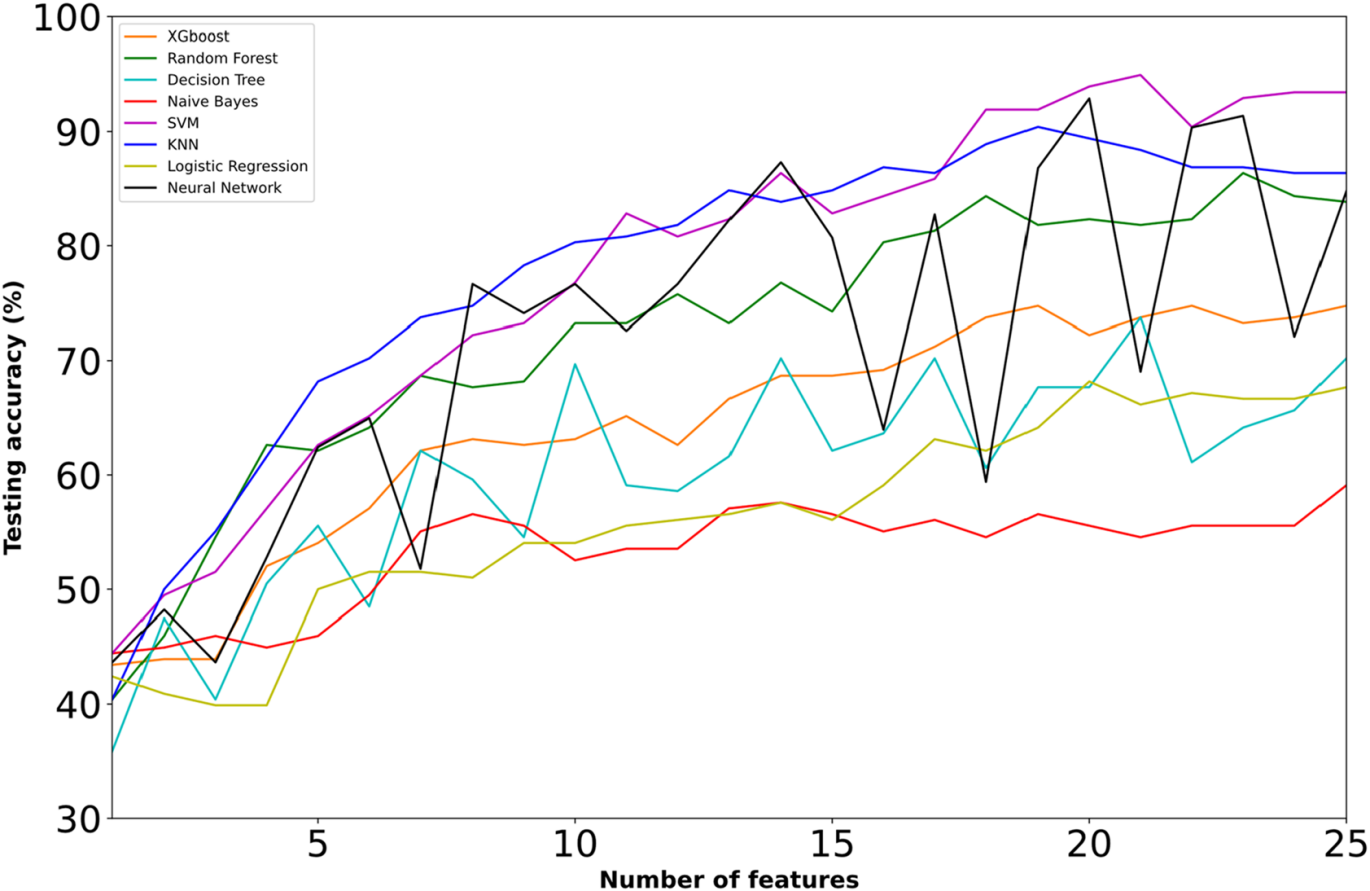
Learning curves with testing accuracy scores for different ML models trained on feature subsets of increasing dimensionality in the 3-class problem (refering to both ACL deficient and ACL reconstructed patients).

Figure 1 demonstrates the accuracy testing performance (%) of the competing ML models with respect to the number of selected features on the 3-class problem. The majority of the ML models had an upward trend in the whole feature dimensionality range, followed by steady testing performance in most of the cases. Specifically, the SVM model showed an upward trend with respect to the first selected features, with a maximum of 94.95% (which was the overall best performance achieved). The second-best accuracy (92.89%) was achieved by the NN model, which presented a non-steadily increasing performance with fluctuations for more than 15 selected features. LR, DTs, RF, XGboost and KNN models also showed an upward trend with moderate accuracies ranging from 68.18% up to 90.40%. In contrast with the other models, Naïve Bayes failed in this task, recording low accuracy testing performances (in the range of 44.44–59.09%).

Table 1 summarizes the results of XGboost, Random Forest, Decision Trees, Naive Bayes, SVM, KNN,Logistic regression and NN on the three-class problem. The number of selected biomechanical parameters, on which the highest accuracy was obtained, lies within the range of 19–23 for the majority of the ML models (in seven out of the eight), whereas the overall maximum performance was achieved by SVM on a group of twenty-one selected (21) biomechanical parameters. Naive Bayes selected less features (14) leading to low accuracy (57.58%). Furthermore, the second-highest accuracy was achieved by NN (92.89%), whereas lower accuracies were obtained by RF, DTs and XGboost (less than 74.75%). Apart from being the most accurate overall, the SVM model recorded the best performance in all the metrics, namely precision (92.16–96.72%), recall (92.19–97.62%) and f1-score (93.07–96.47%).

**Table 1:** Best testing accuracies (%) achieved for ML models in 3-class problem along with precision, recall, f1-score and the optimum number of features.

Models	Accuracy	Classes	Precision	Recall	F1-Score	Num. of features
XGBoost	74.75	CON	70.80	95.24	81.22	19
		ACLD	81.48	44.00	57.14	
		ACLR	79.31	71.88	75.41	
Random Forest	86.36	CON	80.00	95.24	86.96	23
		ACLD	90.00	72.00	80.00	
		ACLR	94.83	85.94	90.16	
Decision Trees	73.74	CON	76.67	82.14	79.31	21
		ACLD	72.09	62.00	66.67	
		ACLR	70.77	71.88	71.32	
Naive Bayes	57.58	CON	65.69	79.76	72.04	14
		ACLD	40.91	54.00	46.55	
		ACLR	66.67	31.25	42.55	
SVM	94.95	CON	95.35	97.62	96.47	21
		ACLD	92.16	94.00	93.07	
		ACLR	96.72	92.19	94.40	
KNN	90.40	CON	85.26	96.43	90.50	19
		ACLD	95.12	78.00	85.71	
		ACLR	95.16	92.19	93.65	
Logistic Regression	68.18	CON	70.64	91.67	79.79	20
		ACLD	57.50	46.00	51.11	
		ACLR	71.43	54.69	61.95	
NN	92.89	CON	96.30	92.86	94.55	20
		ACLD	90.57	96.00	93.20	
		ACLR	90.48	90.48	90.48	

### Explainability to quantify the features’ contribution

In this section, we interpret the contribution of the biomechanical parameters in shaping the AI model’s output. To cope with this, we used explainability analysis on the best performing ML model (SVM). Initially, we performed a global investigation on the 3-class problem to quantify the overall features’ contribution to the problem. Then, we performed explainability analysis on each one of the three trained binary (one-versus-one) SVM models that constitute the 3-class problem. Specifically, we applied SHAP analysis into the following three problems: (i) control group versus ACLD group (local problem 1), (ii) control group versus ACLR group (local problem 2), and (iii) ACLD group versus ACLR group (local problem 3).

**Figure 2:**
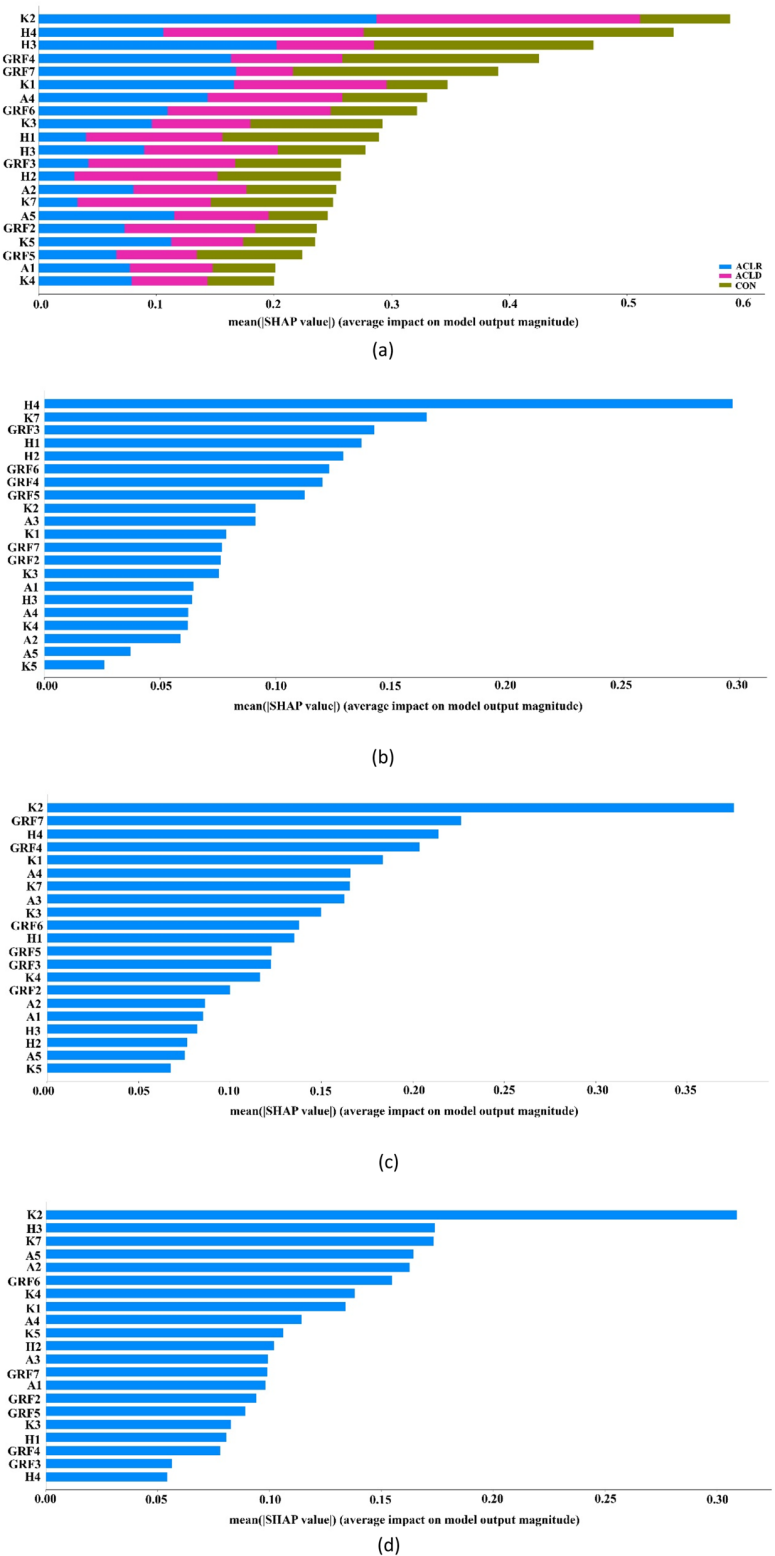
(**a**) Average feature impact magnitude for all instances in the 3-class problem; (**b**) Features’ impact on SVM model output for local problem 1. This figure shows the average impact magnitude for all instances in the task of differentiating the control group vs pre-surgery group; (**c**) Average feature impact magnitude for all instances in the local problem 2 (control versus ACLR); (**d**) Average feature impact magnitude for all instances for local problem 3 (pre-surgery group versus post-surgery group).

Figure 2a visualises the impact of the feature across all classes and the features were sorted by the sum of their SHAP values magnitudes across all instances. In this approach K2, H4, A3, GRF4, GRF7, K1, A4 and GRF6 were the parameters that affected the model output with mean SHAP values higher than 0.3.

Figure 2b depicts the mean absolute value of the SHAP values which represents the SHAP global feature importance for local problem 1 (CON versus the ACLD). It should be noted that the features H4, K7, GRF3, H1, H2 were the most important variables that significantly affected the prediction output. It is also observed that the contribution of H4 is 0.3 while the second-best parameter (K7) and all the remaining ones are below 0.18. From the above, H4 significantly contributes to the separation between the CON group and the ACLD group.

Figure 2c depicts the mean absolute value of the SHAP values for local problem 2 that focuses on the discrimination of the CON and ACLR groups. Features K2, GRF7, H4, GRF4 and K1 were the most important variables that significantly affected the prediction output for the certain groups. Specifically, K2 records a much higher mean absolute value (higher than 0.35) compared to the rest of the features (that exhibit values less than 0.23).

The most important variables that significantly affected the prediction output in the local problem 3 (ACLD group versus ACLR group) were K2, H3, K7, A5 and A2, as shown in Fig. 2d. Similarly to local problem 2, parameter K2 is again the most important separation factor between individuals from the ACLD group and the ACLR group.

### Statistical analysis to identify significant differences between selected features

Statistical comparisons were also performed to identify whether there exist statistically significant differences between the classes considered for the most important features as they have been highlighted by the explainability analysis. Table 2 demonstrates the results of the one-way ANOVA tests, which performed to quantify these differences at the global level (with all three classes considered). As observed, there were statistically significant differences between the group means for all the comparisons.

**Table 2:** Statistical comparison at the global level.

Features	Statistical comparison	CON	ACLD	ACLR
		Mean (std)	Mean (std)	Mean (std)
K2	P = 0.004	8.59 ± 3.81	8.35 ± 4.89	9.72 ± 4.70
H4	P = 0.007	37.93 ± 4.93	36.26 ± 6.42	37.03 ± 5.53
A3	P = 0.000	13.81 ± 6.52	15.91 ± 7.43	16.88 ± 7.25
GRF4	P = 0.000	19.61 ± 4.30	16.94 ± 5.17	16.76 ± 4.84
GRF7	P = 0.000	5.19 ± 1.65	5.81 ± 2.03	6.18 ± 2.92
K1	P = 0.001	21.62 ± 5.88	19.73 ± 6.56	19.88 ± 6.27
A4	P = 0.011	0.18 ± 0.08	0.19 ± 0.08	0.20 ± 0.09
GRF6	P = 0.004	5.69 ± 1.43	6.01 ± 2.21	6.36 ± 2.97

Then we performed statistical comparisons at the local level putting emphasis on the following tasks: (i) ACL diagnosis and (ii) rehabilitation after surgery. Initially, we run independent t-test analysis between the CON and the ACLD groups for the first eight significant biomechanical parameters, which were indicated by the explainability analysis of the specific binary problem (local problem 1). Subsequentially, we employed independent t-test analysis between the control and the ACL-reconstructed groups on the same parameters to identify which of them were modified and/or restored to their normal state (control level) as a measure of evaluating the postoperative progress.

Table 3 summarizes the results of the statistical analysis at the local level. The following remarks can be drawn from Table 5: (i) Significant differences were observed between CON and ACLD for half of the features considered, specifically the first three (H4, K7 and GRF3) along with GRF4; (ii) Four of the parameters (H1, H2, GRF6 and GRF5) that were considered important by the explainability analysis had no significant changes between CON and ACLD groups.

**Table 3:** Statistical analysis at the local level for ACL diagnosis and rehabilitation.

Features*	CON vs ACLD	CON vs ACLR
H4	P = 0.002	P = 0.057
K7	P = 0.000	P = 0.001
GRF3	P = 0.000	P = 0.090
H1	P = 0.288	P = 0.792
H2	P = 0.723	P = 0.326
GRF6	P = 0.061	P = 0.001
GRF4	P = 0.000	P = 0.000
GRF5	P = 0.721	P = 0.147

## Discussion

This paper focuses on the development of a novel approach, which combines an explainable ML-empowered methodology and statistical analysis, for identifying important parameters associated with ACL injury. The problem has been coped as a three-class classification task where the participants of the study were divided into three groups (CON, ACLD and ACLR group). In addition to the classification part, the main contributions of this paper are: (i) to investigate how much each of the features contributed to the final ML decisions, (ii) to estimate the feature importance in the classification process and (iii) to investigate differences in three dimensional GRFs, sagittal plane kinematics and kinetics of the gait cycle for the CON, ACLD and ACLR groups.

Being effective in problems with strong dependencies between features, the ReliefF algorithm was applied to serve as a FS technique and thus reduce the dimensionality of the initial feature space. Eight ML models were employed to perform the 3-class classification task on the reduced feature space where accuracies up to 94.95% were achieved. Specifically, the SVM model had the best performance and it showed an upward trend with respect to the first selected features, with a maximum of 94.95% at 21 features (which was the overall best performance achieved). Furthermore, the SVM model achieved rates from 92.16% up to 97.62% in each class for the metrics precision, recall and f1-score.

Having selected the most accurate ML model, this study attempted to uncover the rationale behind the decision-making mechanism of the trained model and therefore provide an alternative and a more holistic approach of quantifying the contribution of the input biomechanical parameters in the classification process. Specifically, explainability analysis was applied on the best performing ML model (SVM) and a global investigation was initially performed on the 3-class problem to quantify the overall features’ contribution to the problem. As observed K2, H4, A3, GRF4, GRF7, K1, A4 and GRF6 were the most important biomechanical parameters that affected the model output. In order to estimate the feature importance separately, we also performed explainability analysis on each one of the three trained binary (one-versus-one) SVM models that constitute the 3-class problem. Specifically, we applied SHAP analysis into the following three problems: (i) CON group versus ACLD group (local problem 1), (ii) CON group versus ACLR group (local problem 2), and (iii) ACLD group versus ACLR (local problem 3). As observed, in the local problem 1 the main biomechanical parameters were H4, K7 and GRF3. Furthermore, K2, GRF7 and H4 have the main contribution in local problem 2. In addition, from the third local problem K2, H3 and K7 have occurred as the most important biomechanical parameters. Previous studies have observed altered gait biomechanics in the ACL deficient and ACL reconstructed patients compared to healthy individuals^10,11^. These findings may indicate that the employed rehabilitation protocols fail to restore normal walking biomechanics, resulting in aberrant movement patterns. Several of the most important biomechanical parameters of ACL injury diagnosis highlighted by the global as well as the local explainability analysis used in our study coincide with the biomechanical outcomes reported in the literature to be related to altered gait patterns following ACLR. For example, maximum knee extension moment during stance phase (K7) significantly affected the prediction output in both of the aforementioned local problems examined in our study. K7 has been extensively investigated following ACLR and it has been consistently identified as a biomechanical parameter that is deceased following surgery and it is associated with poorer knee function in ACLR patients compared to healthy individuals^11,38^. Additionally, minimum knee flexion angle during stance phase (K2) which had the most important contribution in local problem 2 and a significant one in local problem 3 has been reported to differentiate gait patterns between ACLR and healthy individuals up to 48 weeks post-surgery^38^.

Besides explainability analysis, conventional statistical analysis was further performed to determine whether there exist significant differences between the three groups of our study for the aforementioned selected biomechanical parameters. As it was observed, in most of the cases the outcomes of the explainability and statistical analyses coincide. However, no significant differences were identified for many of those important parameters as shown in the case of local problem 1 (ACL diagnosis) in which H1, H2, GRF6 and GRF5 were identified as important by SHAP whereas their distributions had no significant differences between CON and ACLD. This finding implies that the proposed explainable ML methodology goes beyond the way that traditional statistics work. Features, that would have been neglected by the traditional statistical analysis, are highlighted as contributing parameters that have a significant impact on the ML model’s output when they are combined with other statistically important ones. Moreover, as a measure of evaluating the postoperative progress, we performed statistical analysis for the local problem 1 and local problem 3 on the same parameters to identify which of them were modified and/or restored to their normal state (control level) after the surgery. Two (H4 and GRF3) of the three most important parameters (H4, GRF3, the third parameter) were restored to their initial state after the surgery having no significant differences in the comparison between CON and ACLR groups. This means that these two biomechanical parameters (H4 and GRF3) were initially modified after the ACL injury and they were subsequently restored to their initial state after the surgery.

The clinical significance of our novel approach discussed in this work, which is based on a combination of an explainable ML-empowered methodology and statistical analysis to identify biomechanical parameters during walking associated with ACL injury, should be considered with caution. This can be attributed to the fact that even though gait biomechanics are altered following ACLR, few biomechanical parameters demonstrate consistent results across studies and various tasks^10^. Factors such as, differences in the ACLR techniques (e.g. graft type), individual coping strategies among participants during walking, variations in employed rehabilitation protocols and gender differences may affect gait biomechanics alterations following ACLR as well as their clinical interpretation^10,11,39^.

Explainability via SHAP or other similar tools is a crucial enabler allowing humans to better comprehend the decisions generated by black box models. However, SHAP is limited to simple explanations mainly quantifying the impact of individual features to the models' output^40^. Thus, the inner workings of the trained models and the way that the features are combined to reach the final decision remain hidden. Future work includes the combined use of graphical modelling with well-known explainability tools with the goal of identifying the relationships between features and the possible direct and indirect effect of features to the models’ output. Such graphically-given explanations would enhance our understanding of the real rationale behind the decision-making mechanism of ML-empowered models acting on the tasks of ACL diagnosis and rehabilitation.

## Summary

An explainable ML-empowered methodology was designed, implemented and tested in this paper to identify important biomechanical parameters associated with ACL injury. The proposed extensive experimental setup included gait biomechanical data, a thorough comparative analysis with seven well-known classifiers and a state-of-the-art explainability analysis. According to the findings of the comparative analysis, a 94.95% classification accuracy was achieved by SVM on a group of twenty-one biomechanical parameters. The nature of the selected parameters along with their impact on the prediction outcome (via SHAP) were discussed to uncover the rationale behind the decision-making mechanism of the trained model and therefore provide an alternative and a more holistic approach of quantifying the contribution of the input parameters in the diagnosis of ACL injury. Statistical analysis was further performed to determine whether there exist significant differences between ACL deficient, ACL reconstructed and healthy individuals for the aforementioned parameters. Understanding the contribution of gait biomechanics is a valuable tool for creating more powerful and non-invasive prognostic tools in the hands of physicians, that will point out abnormal gait patterns in patients after ACLR to modify the rehabilitation protocol and avoid the development of osteoarthritis.

## Methods

### Participants

A total of 151 subjects volunteered to participate in this study. Three different groups were defined: (i) ACL-deficient prior to surgery (ACLD), (ii) ACL-reconstructed (ACLR) and (iii) control (CON) group. Inclusion criteria were as follows: (1) all subjects could be of either gender between 18–50 years old and were moderately active, participating in regular activity at least two times per week; (2)The ACLD subjects had suffered a unilateral ACL injury confirmed by an orthopedic surgeon as well as via magnetic resonance imaging and participated in the present study an average of 30 days after injury, but before surgery; (3)The ACLR subjects had a unilateral ACL reconstruction and participated in the present study at least 6 months post-surgery; (4) Individuals with different graft types (i.e., hamstring tendon and patellar tendon grafts) were included in the ACLR group; (5) Both ACLD and ACLR subjects had a healthy contralateral knee and reported no other history of serious lower limb injury, such as bilateral ACL injury or injury to the meniscus, posterior cruciate ligament, medial or lateral collateral ligament in either knee; 6) had been cleared to resume their physical activity at the time of the measurement. The CON subjects were matched for age, gender, and physical activity status and had no history of ACL injury and neurologic disorder or other lower extremity injuries within 12 months prior to participating in the study. Prior to participation, all subjects signed a consent form, and all procedures were approved by the University of Thessaly ethics committee (approval code: 1660). The subjects’ characteristics are presented in detail in Table 4.

**Table 4:** Subjects’ characteristics.

Characteristics	ACLD	ACLR	CON
Gender	31 males and 13 females	40 males and 14 females	34 males and 19 females
Height	175.3 ± 0.86 cm	177.6 ± 0.80 cm	174.1 ± 0.98 cm
Weight	77.38 ± 14.91 kg	76.37 ± 14.35 kg	72.23 ± 15.81 kg

### Testing procedure and data collection

Upon entering the gait laboratory, the subjects received instructions regarding the testing procedure and were familiarized with the walking task. ACLD and ACLR subjects completed the subjective Knee injury and Osteoarthritis Outcome Score (KOOS) evaluation form, which is considered a reliable measure of 5 outcomes, including activities of daily living, sport and recreation, pain, and knee-related quality of life^41^. Anthropometric measurements were recorded, and 20 spherical retroreflective markers were positioned bilaterally on anatomic landmarks and specific locations of the pelvis and lower limbs according to the marker set described in the literature^42,43^. Subsequently, the subjects walked barefoot along the 10 m laboratory walkway within ± 5% of their individual self-selected walking speed (SWS). SWS was measured during familiarization using infrared timing gates located in the middle of the walkway and was maintained throughout data collection via a metronome. Trials were performed until at least 5 complete gait cycles were recorded with each foot (left and right side) landing on the force platform. A trial was considered valid if the foot of the side being tested made a clean contact with the force platform located in the middle of the walkway and the walking speed was within ± 5% of the individual SWS. Kinematic data were collected using 10 optoelectronic cameras (Vicon T-series, Oxford, UK) at 100 Hz and kinetic data were collected at 1000 Hz via a force platform (Bertec 4060–10, OH) embedded in the floor synchronized with the kinematic data.

### Data analysis

**Figure 3:**
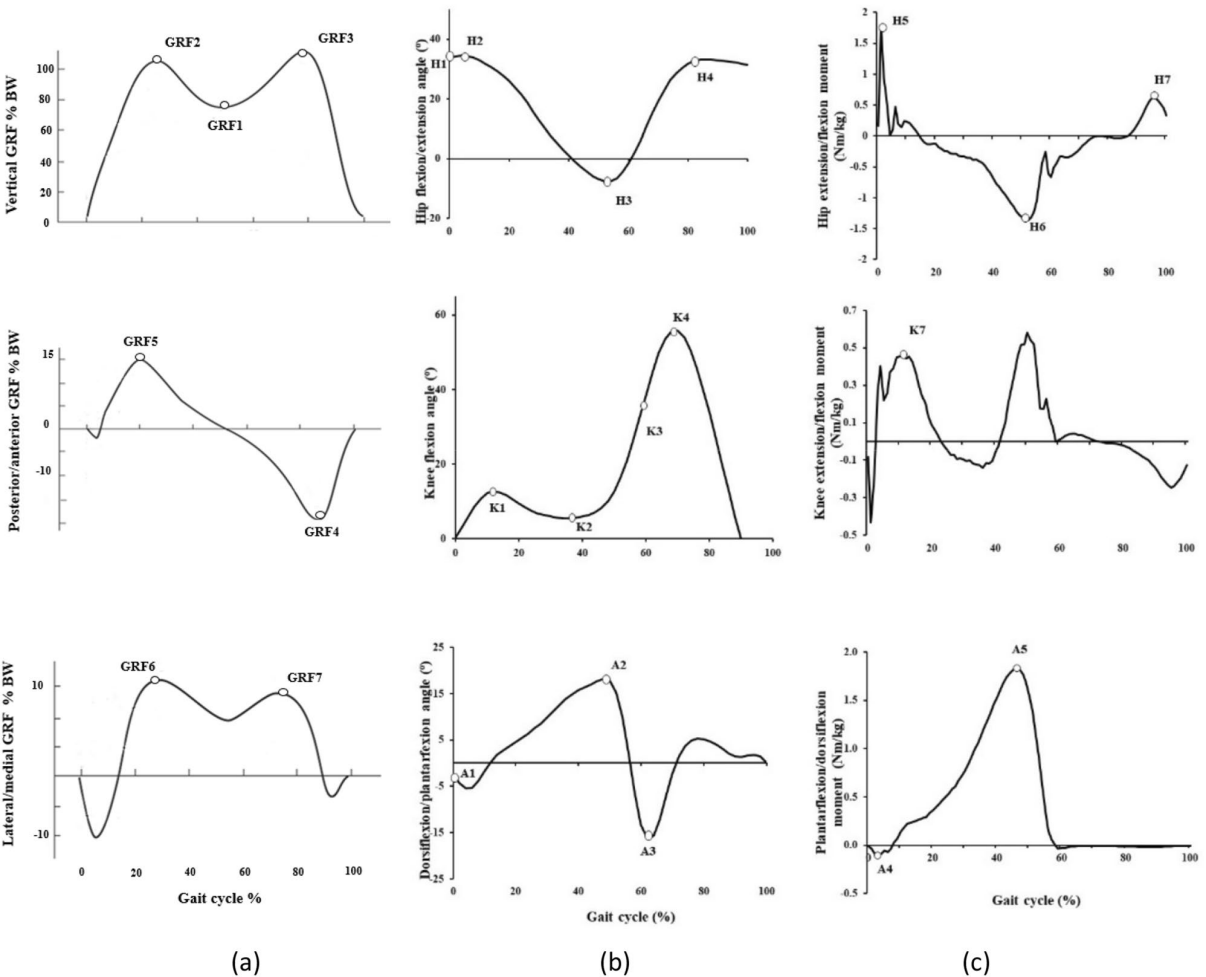
Three dimensional GRFs (**a**), sagittal plane kinematic (**b**) and kinetic (**c**) variables of interest during walking. Ankle dorsiflexion, knee flexion, hip flexion, anterior and medial GRFs, as well as internal ankle plantar flexion, knee extension and hip extension moments were all defined as positive.

The symmetrical center of rotation estimation (SCoRE)^44^ and the symmetrical axes of rotation approach (SARA)^45^ were applied to optimize the calculation of the hip joint center and knee joint flexion axis, respectively. The initial contact and toe-off events of stance phase were determined from the vertical GRF (20 N threshold) and the subsequent ipsilateral initial contact was determined from motion data using the Vicon Nexus software. Kinematic and GRF data were lowpass filtered with a 4th order Butterworth filter at 10 and 40 Hz, respectively. The SCoRE and SARA approach were combined with the Plug-In-Gait model to generate motion features according to the Nexus Advanced Gait Workflow (Nexus 2.10, Vicon Metrics Group Ltd, Oxford, UK). Inverse dynamics were used combining inertia properties of the segments as well as sagittal plane kinematic and GRF data to calculate net joint moments of the lower limbs during the gait cycle. GRFs were expressed as a percentage of body weight, while net joint moments were expressed as internal moments and were normalized to body mass. Selected gait variables were extracted for each trial of each subject. A total of 155 trials were analysed for ACLD group, 204 trials for ACLR group and 298 trials for CON group, respectively. The three-dimensional GRFs, sagittal plane kinematic and kinetic variables of interest are presented in Fig. 3 and Table 5. Data were analyzed from the ACLD/ACLR subjects’ involved limb and for the control subjects, this was randomly assigned.

**Table 5:** Evaluated parameters of the gait cycle for vertical and horizontal GRFs and sagittal plane kinematics and kinetics.

Variables	Description
GRF1	Local minimum vertical GRF during support (% BW)
GRF2	First vertical GRF peak (% BW)
GRF3	Second vertical GRF peak (% BW)
GRF4	Anterior (propulsive) GRF peak (% BW)
GRF5	Posterior (braking) GRF peak (% BW)
GRF6	First medial GRF peak (% BW)
GRF7	Second medial GRF peak (% BW)
H1	Hip flexion angle at initial contact (°)
H2	Maximum hip flexion angle during stance phase (°)
H3	Maximum hip extension angle during stance phase (°)
H4	Maximum hip flexion angle during swing phase (°)
H5	Maximum hip extension moment during stance phase (Nm/kg)
H6	Maximum hip flexion moment during stance phase (Nm/kg)
H7	Maximum hip extension moment during swing phase (Nm/kg)
K1	Peak knee flexion angle during stance phase (°)
K2	Minimum knee flexion angle during stance phase (°)
K3	Knee flexion angle at foot off (°)
K4	Maximum knee flexion angle during swing phase (°)
K5	Knee flexion angle at first maximum knee extension moment during stance phase (°)
K6	Knee flexion angle at first vertical ground rection force peak (°)
K7	First maximum knee extension moment during stance phase (Nm/kg)
A1	Ankle angle at initial contact (°)
A2	Maximum dorsi-flexion angle during stance phase (°)
A3	Maximum plantar-flexion angle over the entire gait cycle (°)
A4	Maximum dorsiflexion moment during stance phase (Nm/kg)
A5	Maximum plantarflexion moment during stance phase (Nm/kg)

### Machine learning workflow

In order to identify knee kinematics associated with ACL injury, we designed, implemented and tested a multi-stage ML pipeline. Data were normalised to [0, 1] to build a common basis for the feature selection (FS) and the ML estimators. To rank our biomechanical parameters a well-established FS technique was applied. ReliefF algorithm^46^ is a supervised learning algorithm and it is such effective in problems where strong dependencies between features are observed. Various well-known ML classifiers were evaluated for their suitability. Hyperparameter selection was implemented to avoid bias error, overfitting and optimize the performance of our ML models. Specifically, we used XGboost algorithm^47^ and Random Forest (RF)^48^, which are ensemble learning algorithms and they are used due to their fast execution speed and increased model performance. Decision trees (DTs) were also evaluated providing a more interpretable decision-making mechanism^49^. Furthermore, we tested Naïve Bayes algorithm^50^, which is based on applying Bayes’ theorem and this method can be extremely fast. Being effective in high-dimensional spaces, SVM algorithms were also Included in our experimental analysis^51^. Moreover, Logistic Regression (LR)^52^ and the K-Nearest Neighbor (KNN) algorithm^53^ were tested. LR was employed to set the baseline performance obtained by a linear model and KNN was selected due to its ability to deal with the overfitting problems that appear in high-dimensional spaces. Neural networks (NN) were also tested because they can handle complex data using a human brain inspired structure that mimics the way that biological neurons communicate to each other^54^. Different activation functions were tested (including tanh, sigmoid and ReLU) for their suitability as part of the hyperparameter optimisation phase.

For the evaluation of the proposed classifiers, a stochastic 70–30% random data split was applied to generate the training and testing subsets, respectively^55^. Specifically, the learning was performed on the stratified version of the training sets and the final performance was estimated on the accuracy testing sets. Furthermore, the performance of the classifiers was also evaluated in terms of the recall (or sensitivity), f1-score and precision as additional evaluation criteria^56^.

In this paper, we also: (i) investigated how much each of the features contributed to the final decision and (ii) estimated the feature importance. In order to achieve this, we used SHapley Additive exPlanations (SHAP) which are based on Shapley Values of game theory^57,58^. SHAP offers the ability to interpret ML models, which are often treated as black boxes. In this paper, we employed SHAP to rank features in terms of their impact on the final ML outputs and to build a mini explainer model. This enhances our understanding of the internal decision-making rationale of the trained AI models especially with respect to the mechanism with which selected biomechanical parameters are combined to produce decisions on ACL diagnosis and rehabilitation.

### Statistical analysis

One-way analysis of variance (ANOVA) was used to investigate differences in sagittal plane kinematics and kinetics of gait cycle for the CON, ACLD and ACLR groups^59^. Furthermore, independent sample t-tests were employed to compare the first eight significant biomechanical parameters between the CON and the ACLD groups, which were indicated by the explainability analysis. On the same parameters, independent sample t-tests were also employed to evaluate the postoperative progress^60^. The significance level in our statistical comparisons was set at P < 0.05.

### Institutional review board statement

The study was conducted according to the guidelines of the Declaration of Helsinki and approved by the Ethics Committee of the University of Thessaly (protocol code 1660 and date of approval 03/06/2020).

### Informed consent

Informed consent was obtained from all subjects involved in the study.

## Data Availability

The dataset generated during and/or analysed during the current study are not publicly available, but are available from the corresponding author on reasonable request.
